# Editorial: Trace elements and aquatic plants: accumulation, ecological impact, and biomonitoring applications

**DOI:** 10.3389/fpls.2025.1600036

**Published:** 2025-04-30

**Authors:** Ludmiła Polechońska, Agnieszka Klink

**Affiliations:** Department of Ecology, Biogeochemistry and Environmental Protection, University of Wrocław, Wrocław, Poland

**Keywords:** abiotic stress, macrohydrophytes, macroalgae, toxic metals, synergistic and antagonistic effects

Aquatic plants (macrohydrophytes) form a greatly variable group of plants, in terms of their life form, ecology and habitat preferences. The group includes permanently or periodically submerged, floating or emergent plants (mosses, ferns and angiosperms) and filamentous macroalgae (Pokorny and Kvet, 2003; Wetzel, 2001). They are the major components of most types of aquatic ecosystems playing a significant role in shaping the physical and chemical environment as well as establishing the structure and functioning of biocenosis. To mention only their most important functions, macrohydrophytes provide a considerable number of ecological niches and, as primary producers, constitute the basis of the food web. They also have the ability to absorb and accumulate numerous chemical substances from water and/or bottom sediments, including trace elements. Therefore, they contribute to regulating nutrient availability and controlling water eutrophication and purification, which can be utilized in phytoremediation. Importantly, aquatic plants are recognized as bioindicators and biomonitors because they respond to a variety of environmental conditions and pollutants (De et al., 2019; Chaurasia, 2022).

Macrohydrophytes are recognized bioaccumulators which can bioconcentrate trace elements in their tissues up to thousands of ppm and even 1000-fold the element concentration in the habitat (Yang and Ye, 2009; Tanwir et al., 2020). The uptake of trace elements is regulated by biological factors such as degree of plant development, morphology, genetic characteristics, as well as phenophase and non-biological factors such as exposure time, redox, Eh, pH, salinity, temperature, organic matter content, metal form and concentration in water and/or sediment (Lewis, 1995; Yang and Ye, 2009; Krems et al., 2013). Low concentrations of some trace elements (e.g., Mn, Fe, Co, Cu, Zn, Ni) are vital and essential for normal plant metabolism and development (Maleva et al., 2012). As Szabó et al. reported in this Research Topic, Mn and Fe deficiency leads to reduced total chlorophyll content and limited growth of *Lemna gibba*. On the other hand, in case of too high concentrations of trace elements in habitat, the bioaccumulation in macrohydrophyte tissues may not equate with the physiological benefits of the element and reach toxic levels causing stress responses (Krems et al., 2013; Tanwir et al., 2020).

The negative effect of toxic trace elements is often associated with excessive production of reactive oxygen species (ROS), which induce oxidative stress (Maleva et al., 2012). The overproduction of ROS cause detrimental changes in the ultrastructure of plant cells and disruption of physiological and biochemical processes (Nayek et al., 2010; Saqira et al., 2024) such as cell respiration, photosynthesis, and nitrogen metabolism. Reactive oxygen species can damage enzymes, proteins, DNA, and lipids. They also cause degradation of photosynthetic pigments, the ultrastructure of the chloroplast and the assembly of plastoglobuli, changes in vacuoles (Tanwir et al., 2020), and impairment of the membrane selective permeability which results in cell plasmolysis (Basile et al., 2012). All these disruptions lead to decrease of cell division and retardation of plants growth. Common morphological and anatomical modifications due to trace element stress are therefore a reduced number of lateral roots and root hairs; changes in root diameter, epidermal cells, parenchymatous cells, central vein, and conductive tissue, as well as change in stem and leaf thickness; reduced number of leaves and leaf area (Yadav et al., 2021; Yang and Ye, 2009). In this Research Topic, Asaeda et al. reported a strong decrease in chlorophyll concentration in *Elodea densa* exposed to high concentrations of Fe, while Huang et al. observed a discoloration of the macroalgae *Gracilaria bailiniae* thalli and a decrease in photosynthetic efficiency due to exposure to high Cd concentrations. Importantly, in the study by Huang et al. numerous injuries to the ultrastructure of cortical cells were observed, such as vacuolization due to increase in the cell wall thickness, degeneration of chloroplasts, increased number of plastoglobuli, disrupted and swollen mitochondria, and reduced number of starch grains. Plants usually resist oxidative stress induced by trace elements by increasing the activity of antioxidant enzymes (e.g., peroxidase, catalase, superoxide dismutase) (Nayek et al., 2010; Sridhar et al., 2020). Interestingly, in the current Topic Asaeda et al. showed that excess Fe in the environment causes a decrease in the level of some antioxidant enzymes such as catalase and ascorbate peroxidase in the tissues of *E. densa*, and consequently an increase in H_2_O_2_ accumulation. Since the level of these ROS in *E. densa* positively correlates with the Fe content in the environment, it may be an important indicator of the level of environmental stress.

Some species exhibit defense mechanisms to prevent intoxication and oxidative stress, such as the reduction of uptake (Tanwir et al., 2020) or the inactivation of toxic ions (Basile et al., 2012; Nayek et al., 2010). The toxic effects can also be modified by synergistic or antagonistic interactions with other abiotic and biotic stressors to which macrophytes are usually simultaneously exposed in their natural habitats (Polechońska and Samecka-Cymerman, 2018; Gaudard et al., 2018). In this context, Asaeda et al. noted that the combined effects of high Fe concentrations in water and high photosynthetically active radiation (PAR) intensity was lethal to *E. densa* because Fe destroyed activity of antioxidant enzymes, which was not observed when the factors were applied separately. Also, Szabó et al. observed that the negative effects of nutritional metals (Fe, Mn) deficiency on the chlorophyll content of *L. gibba* were enhanced by a high pH of the water. On the other hand, Mazacotte et al. found out that elevated temperature reduced the sensitivity of submerged macrohydrophytes to the potentially harmful agricultural runoff enriched with trace elements, among others. Similarly, Huang et al. reported interactions between two trace metals (La and Cd) and showed that La mediates the production of flavonoids and lipids in the tissues of *G. bailiniae*, and thus plays a protective role against Cd. It limits its negative impact on, photosynthetic pigments content, photosynthetic efficiency and overall plant growth, among others.

The presented Research Topic contributes to the explanation of the interactions between aquatic plants and trace elements and thus supports their use in phytoremediation, biomonitoring of environmental pollution, wastewater treatment and many other eco-environmental technologies and applications.
